# The Prognosis of Breast Cancer Patients after Mastectomy and Immediate Breast Reconstruction: A Meta-Analysis

**DOI:** 10.1371/journal.pone.0125655

**Published:** 2015-05-29

**Authors:** Xue Yang, Chenfang Zhu, Yan Gu

**Affiliations:** Department of General Surgery, Shanghai Ninth Hospital affiliated with Shanghai Jiaotong University school of Medicine, Shanghai, 200011, China; University of North Carolina School of Medicine, UNITED STATES

## Abstract

**Background:**

An increasing number of patients with breast cancer are being offered immediate breast reconstruction (IBR). The aim of this study was to analyze the impact of IBR on the prognosis of patients with breast cancer.

**Methods:**

We searched the electronic databases of Medline (Pubmed), ISI Web of Knowledge, Embase, and Google Scholar databases for studies reporting the overall recurrence, disease-free survival (DFS), and overall survival (OS) of patients after mastectomy only and mastectomy with IBR. With these data, we conducted a meta-analysis of the clinical outcomes.

**Results:**

Fourteen studies, including 3641 cases and 9462 controls, matched our criteria. Relevant information was extracted from these 14 studies. There was no significant heterogeneity (P for Q-statistic > 0.10 and I^2^ < 25%). Patients who underwent IBR showed no increased risk of overall recurrence of breast cancer (RR = 0.89; 95% confidence interval [CI]: 0.75, 1.04; *P* = 0.14). Furthermore, patients receiving IBR had similar DFS (RR = 1.04; 95%CI: 0.99, 1.08); *P* = 0.10) and OS (RR = 1.02; 95%CI: 0.99, 1.05; *P* = 0.24)) as those of control patients.

**Conclusion:**

This meta-analysis provides evidence that IBR does not have an adverse effect on prognosis. These data suggest that IBR is an appropriate and safe choice for patients with breast cancer.

## Introduction

Breast cancer is now the leading cause of cancer death among women in economically developing countries. This statistic represents a shift from the previous decade, during which the most common cause of death from cancer was cervical cancer[1]. There are approximately 13 million new cases of breast cancer worldwide each year[2]. Mastectomy is the primary treatment for breast cancer; however, it damages a patient’s body image and has adverse effects on emotions, psychology, and social life[3].

To correct these problems, breast reconstruction has become increasingly popular. Patients who have undergone mastectomy have two options for breast reconstruction: immediate breast reconstruction (IBR) and delayed breast reconstruction (DBR). IBR is advantageous over DBR because it decreases the total number surgical procedures and the risks therein. Additional advantages include psychological benefits, reduced recovery time, improvements in the quality of life, and lower costs[4–6]. Some factors, such as age, socio-economic status, and tumor stages, can influence whether patients will receive IBR following mastectomy. These factors also influence the prognosis of the disease[7].

Because IBR is an additional surgical procedure, it may increase postoperative complications (such as flap necrosis, infection, hematoma)[8,9] and delay the initial time to adjuvant chemotherapy in some patients[10,11]. Adjuvant chemotherapy, an important component of systemic therapy for patients with breast cancer, decreases disease recurrence and improves survival. Johnson et al reported that large intervals between surgery and initial chemotherapy can be harmful[12]. Lohrisch et al found no significant differences in recurrence-free survival (RFS) and overall survival (OS) among women who started chemotherapy up to 12 weeks after surgery (groups analyzed: ≤ 4 weeks, > 4 weeks to 8 weeks, > 8 weeks to 12 weeks), but the 5-year OS and RFS of women who started chemotherapy more than 12 weeks after surgery were lower than those of women who started earlier (*P* < 0.05)[13]. These findings suggest that the prognosis of breast cancer may be compromised by delaying chemotherapy for more than 12 weeks after definitive surgery, as might be the case for patients who undergo IBR. Therefore, we systemically reviewed relative data to investigate the impact of IBR on the prognosis of breast cancer.

## Methods

### Search strategy and selection criteria

**Fig 1:**
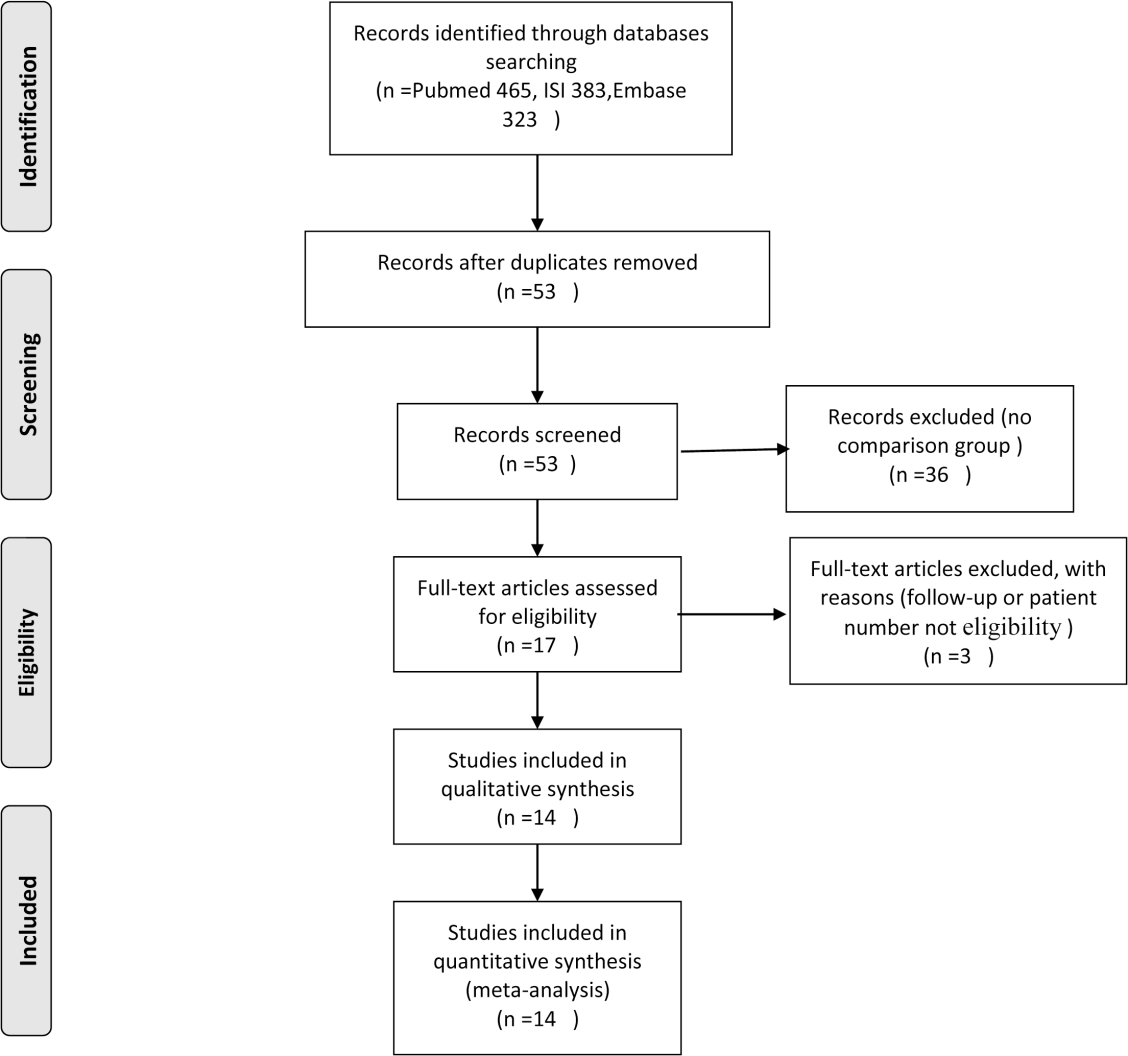
A flow diagram of the search process.

The Medline (Pubmed), ISI Web of Knowledge, Embase, and Google Scholar databases were searched by entering the following terms: “immediate breast reconstruction,” “recurrence,” “survival,” “safety,” and “prognosis.” The search was limited to the English language only. The inclusion criteria were as follows: (1) the study enrolled more than 50 patients; (2) the follow-up was more than 12 months; (3) the type of surgery was not restricted; and (4) the patients had first-time invasive breast cancer. The exclusion criteria were as follows: (1) the results were published in a case report or systematic review; (2) no comparison group was used; or (3) the patients had rare types of tumors, such as phyllodes, sarcoma, or lymphoma. The search covered articles published up to 2014 (the progression of study selection is shown in Fig 1). All of this work was completed independently by two reviewers. If there were different opinions, then a third reviewer was consulted to reach a consensus.

### Quality assessment

The quality of the articles was assessed by using the MINORs (methodological index for non-randomized studies) scale, a validated, 12-item tool with a total of 24-points[14]. Inter-rater reliability was assessed using Cohen Kappa statistic. The level of agreement between the two reviewers is reported with a Kappa value, the interpretation of Kappa as follows: Kappa < 0, less than chance agreement; 0.01–0.20, slight agreement; 0.21–0.40, fair agreement; 0.41–0.60, moderate agreement; 0.61–0.80, substantial agreement; 0.81–0.99, almost perfect agreement[15].

### Data extraction

The following information was extracted from all of the eligible studies: first author’s name and country, year of publication, number of patients enrolled, age, tumor stage, type of reconstruction, type of surgery, follow-up time, recurrence (including local recurrence, locoregional recurrence, and distant recurrence), disease-free survival (DFS) and overall survival (OS).

### Statistical analysis

For the overall recurrence, DFS, and OS of each study, the relative risk (RR) and its 95% CI were used to estimate the association between the different variants. Between-study heterogeneity of the RRs for the same outcome was tested by determining the Q statistic[16]. If *P*>0.10, indicating that there was no significant heterogeneity between studies, then the fixed-effects model was used to calculate the pooled RRs. If the between-study heterogeneity was significant, then a random-effects model was selected. The I^2^ index can reflect the severity of the heterogeneity. An I^2^ value of less than 25% represents low heterogeneity; 25% to 50%, moderate heterogeneity; and more than 50%, high heterogeneity[17].

Publication bias was investigated by constructing a funnel plot, in which the standard error of log (RR) of each study was plotted against its log (RR). If the funnel plot was asymmetric, then Egger’s linear regression was used to test for publication bias. The significance of the intercept was determined by the *t* test, as suggested by Egger, and a *P* value less than 0.05 was considered significant[18]. To test the reliability of the results of the meta-analysis, we made sensitive analysis by excluding a study and comparing the results with or without this study. All analyses were conducted by using Review Manager 5.1 (The Nordic Cochrane Centre, The Cochrane Collaboration, 2011) and SPSS Statistical software (Version 19, SPSS Inc., IBM). The outcomes of the meta-analysis are presented graphically with Forest plots.

## Results

After searching the databases mentioned above, 53 articles received full text review, of which 14 studies met the inclusion criteria. Of these 14 eligible studies, 11 were retrospective cohort studies, including 2 matched-cohort studies, 1 was an historical prospective cohort study, and 2 were prospective cohort studies. The studies included a total of 3641 cases and 9462 controls[19–32]. Patients of 4 studies had skin-sparing or nipple-sparing mastectomy and IBR[20,23,25,27], while the others had mastectomy associated with IBR[19,21–22,24,26,28–32]. Most of the control patients underwent mastectomy; only two study enrolled patients underwent breast conservation surgery or nipple-preserving mastectomy into the control group[20,26]. All studies reported recurrence (including local recurrence, regional recurrence, or distant metastasis), 6 reported DFS, and 8 reported OS. The summary information of the included studies is shown in Tables 1, 2 and 3.

**Table 1:** Demographics of cohort studies included in the meta-analysis.

Author(year published)	Patients/n IBR/Control	Age, yearsIBR/Control	Region of study	Follow-up/mIBR/Control	Adjuvant treatments	Mastectomy type	Reconstruction type	Study Design
**Noguchi[19]**	83/153	82/47.7^a^	Japan	41/58	CT	MT	LDM, TRAM,	Retrospective cohort study
**(1992)**							Implant	
**Yoshimura[20]**	122/92	44/50^b^	Japan	78/55	CT	NMT	NMT +Implant	Retrospective cohort study
**(1996)**								
**Murphy[21]**	158/1262	48/66^b^	USA	75/75	NA	MT	Implant, TRAM,	Retrospective cohort study
**(2002)**							LD+implant	
**Petit[22]**	518/159	69.3/22^a^	Italy	70/71	CT, HT	MT	Implant, TRAM,	Retrospective cohort study
**(2008)**							LD+implant	
**Ueda[23]**	74/178	45.7/55^c^	Japan	50/54	CT, HT, RT	MT	SMIBR+TRAM,	Prospective cohort study
**(2008)**							LD, Implant, DIEP	
**McCarthy[24]**	309/309	46.8/50.8^c^	USA	68.4/68.4	CT, HT, RT	MT	Implant, tissue,	Retrospective cohort study (matched)
**(2008)**							expander	
**Gerber[25]**	108/130	47/58^b^	Germany	101/101	CT, RT	MT	SMIBR+LD,	Historical prospective cohort study
**(2009)**							implant	
**Min [26]**	120/1699	40.7/47.6^b^	Korea	42.1/39.2	CT, RT	BCS	LD	Retrospective cohort study
**(2010)**								
**Lim[27]**	87/810	38.4/47.4^b^	Korea	62.5/65	CT, HT, RT	MT	SMIBR+TRAM,	Retrospective cohort study (matched)
**(2010)**							LD, implant	
**Noguchi[19]**	83/153	82/47.7^a^	Japan	41/58	CT	MT	LDM, TRAM,	Retrospective cohort study
**(1992)**							Implant	
**Yoshimura[20]**	122/92	44/50^b^	Japan	78/55	CT	NMT	NMT +Implant	Retrospective cohort study
**(1996)**								
**Murphy[21]**	158/1262	48/66^b^	USA	75/75	NA	MT	Implant, TRAM,	Retrospective cohort study
**(2002)**							LD+implant	
**Petit[22]**	518/159	69.3/22^a^	Italy	70/71	CT, HT	MT	Implant, TRAM,	Retrospective cohort study
**(2008)**							LD+implant	
**Ueda[23]**	74/178	45.7/55^c^	Japan	50/54	CT, HT, RT	MT	SMIBR+TRAM,	Prospective cohort study
**(2008)**							LD, Implant, DIEP	
**McCarthy[24]**	309/309	46.8/50.8^c^	USA	68.4/68.4	CT, HT, RT	MT	Implant, tissue,	Retrospective cohort study (matched)
**(2008)**							expander	
**Gerber[25]**	108/130	47/58^b^	Germany	101/101	CT, RT	MT	SMIBR+LD,	Historical prospective cohort study
**(2009)**							implant	
**Min [26]**	120/1699	40.7/47.6^b^	Korea	42.1/39.2	CT, RT	BCS	LD	Retrospective cohort study
**(2010)**								
**Lim[27]**	87/810	38.4/47.4^b^	Korea	62.5/65	CT, HT, RT	MT	SMIBR+TRAM,	Retrospective cohort study (matched)
**(2010)**							LD, implant	
**Eriksen[28]**	300/300	48/48^c^	Sweden	144/138	CT, HT, RT	MT	implant	Retrospective cohort study
**(2011)**								
**Nedumpara[29]**	135/452	47/59^c^	England	55/55	CT, HT, RT	MT	LD, implant	Retrospective cohort study
**(2011)**								
**Reddy[30]**	494/427^c^	47.8/56.4^c^	USA	54/54	CT, HT, RT	MT	DIEP, LD, TRAM, SGAP, SIEA, implant	Retrospective cohort study
**(2011)**								
**Lee[31]**	1000/3183	42.2/47.9^b^	Korea	56.4/60	NA	MT	TRAM	Retrospective cohort study
**(2012)**								
**Ota[32]**	133/308	46/58^c^	Japan	47/44	CT, HT, RT	MT	TE	Prospective cohort study
**(2014)**								

^a^ Percentage of patients younger than 50 years

^b^ mean age

^c^ median age.

N:number, m:months, BCS, breast conserving surgery, CT: chemotherapy, DIEP: deep inferior epigastric perforator, HT: hormonal therapy, IBR, immediate breast reconstruction, LD: latissimus dorsi, MT: mastectomy, RT: radiation therapy, S-GAP: superior Gluteus artery perforator, SIEA: superficial inferior epigastric artery flap, SMIBR: skin-sparing/nipple-sparing mastectomy and immediate breast reconstruction, TE: tissue expander, TRAM: transverse rectus abdominis musculocutaneous; NMT: nipple-preserving mastectomy

**Table 2:** Outcome of the cohort studies included in the meta-analysis.

Author	AJCC tumor stage		Recurrence	DFS	OS
**Noguchi[19]**		0-III	IBR:3/83	IBR:75/83	IBR:76/83
			Control:7/153	Control:132/153	Control:140/153
**Yoshimura[20]**		I-II	IBR:14/122	IBR:108/122	IBR:116/122
			Control:9/92	Control:81/92	Control:89/92
**Murphy[21]**		0-IV	IBR:2/158	NA	NA
			Control:9/1262		
**Petit[22]**		I-III	IBR:110/518	IBR:392/518	IBR:464/518
			Control:43/159	Control:110/159	Control:133/159
**Ueda[23]**		0-III	IBR:8/74	IBR:67/74	NA
			Control:19/178	Control:160/178	
**McCarthy[24]**		I-III	IBR:59/309	NA	NA
			Control:74/309		
**Gerber[25]**		0-III	IBR:12/108	NA	IBR:83/108
			Control:15/130		Control:100/130
**Min[26]**		0-IV	IBR:9/120	NA	NA
			Control:133/1699		
**Lim[27]**		IIB-III	IBR:4/87	IBR:61/87	IBR:69/87
			Control:20/810	Control:547/810	Control:607/810
**Eriksen[28]**		I-III	IBR:85/300	NA	IBR:249/300
			Control:98/300		Control:231/300
**Nedumpara[29]**		NA	IBR:12/135	NA	IBR:108/135
			Control:38/452		Control:360/452
**Reddy[30]**		0-III	IBR:11/494	NA	NA
			Control:17/427		
**Lee[31]**		0-III	IBR:18/1000	NA	NA
			Control:38/3183		
**Ota[32]**		NA	IBR:15/133	IBR:118/133	IBR:125/133
			Control:35/308	Control:271/308	Control:290/308

AJCC: American Joint Committee on Cancer, DFS: disease-free survival, IBR, immediate breast reconstruction, NA: not available, OS: overall survival

**Table 3:** Clinical stage of the studies included in the meta-analysis.

Stage^&^	0-I IBR/Control	II IBR/Control	III IBR/Control	IV IBR/Control	Other IBR/Control
Study					
**Noguchi[19]**	25.3/30	57.8/48.4	16.9/21.6		
**Yoshimura[20]**	82.1/79.3	17.9/20.7			
**Murphy[21]**	54.4/44.2	30.4/31	2.5/12.8	1.3/4.1	11.4/7.8
**Petit[22]** *	14.9/8.2	44/41.5	36.9/44		
**Ueda[23]** ^**#**^	55.4/40	44.6/57.8	0/2.2		
**McCarthy[24]**	31.7/31.7	53.1/53.1	15.2/15.2		
**Gerber[25]**	20.4/27	74.1/68	5.5/5		
**Min[26]**	56.7/44	25.8/40.7	10.8/11.5	0/0.3	
**Lim[27]**		9.2/6.7	90.8/93.3		
**Eriksen[28]**	NA				
**Nedumpara[29]**	NA				
**Reddy[30]**	61.1/39.3	23.1/38	8.7/18.7		
**Lee[31]**	53.7/28.1	37.1/46.3	7.7/15.1		
**Ota[32]**	NA				

^&^ The value is percentage

* Grading

^**#**^ Tumor stage

NA not available.

**Fig 2:**
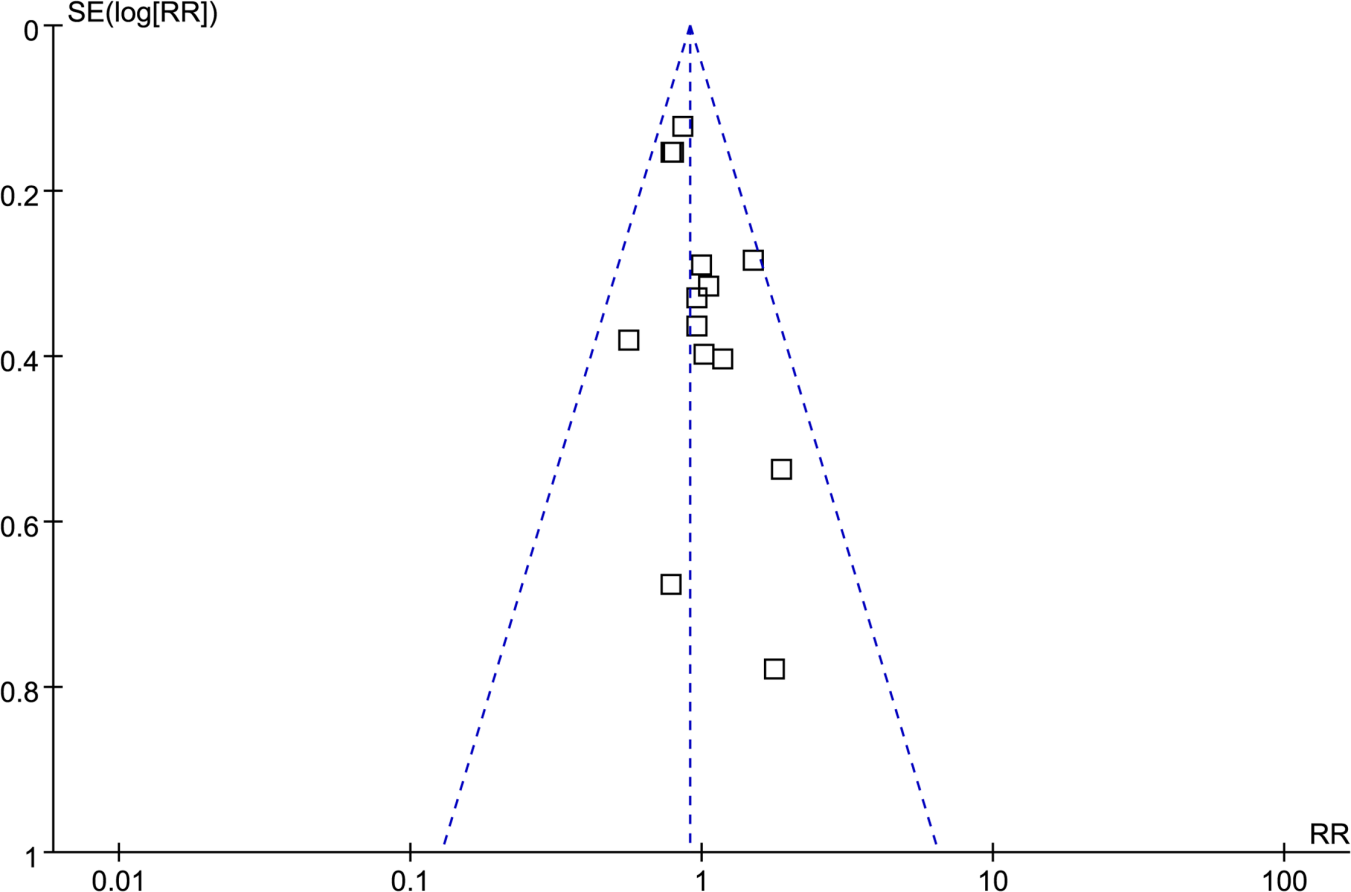
A funnel plot of the 14 included studies.

All 14 studies were independently assessed by 2 of the authors using the MINORs scale. The scores of these studies ranged from 16 to 21 points. The inter-rater reliability was 0.745, which signifies a substantial level of agreement. A funnel plot of the studies is symmetric, which demonstrates that there is no apparent publication bias (Fig 2).

**Fig 3:**
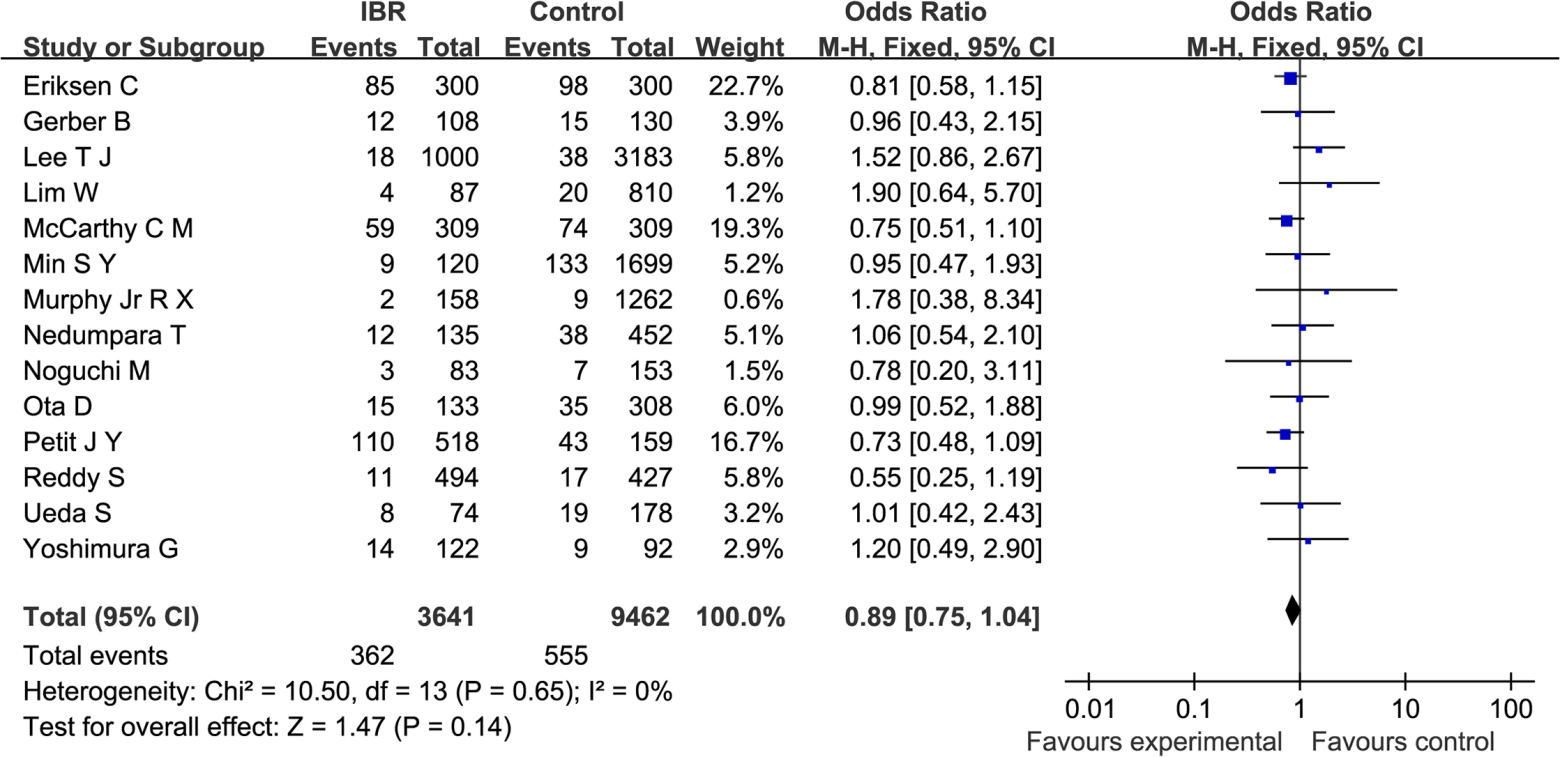
A Forest plot of the pooled RR of recurrence for the IBR and Control groups.

First, overall recurrence was examined across the 14 studies; no significant heterogeneity was found (Q statistic: *P* = 0.65; I^2^ = 0%). The pooled RR for overall recurrence was 0.89 (95%CI: 0.75, 1.04). As shown in Fig 3, the results indicate that there was no significant difference in cancer recurrence between patients who underwent IBR and the control patients (*P* = 0.14).

**Fig 4:**
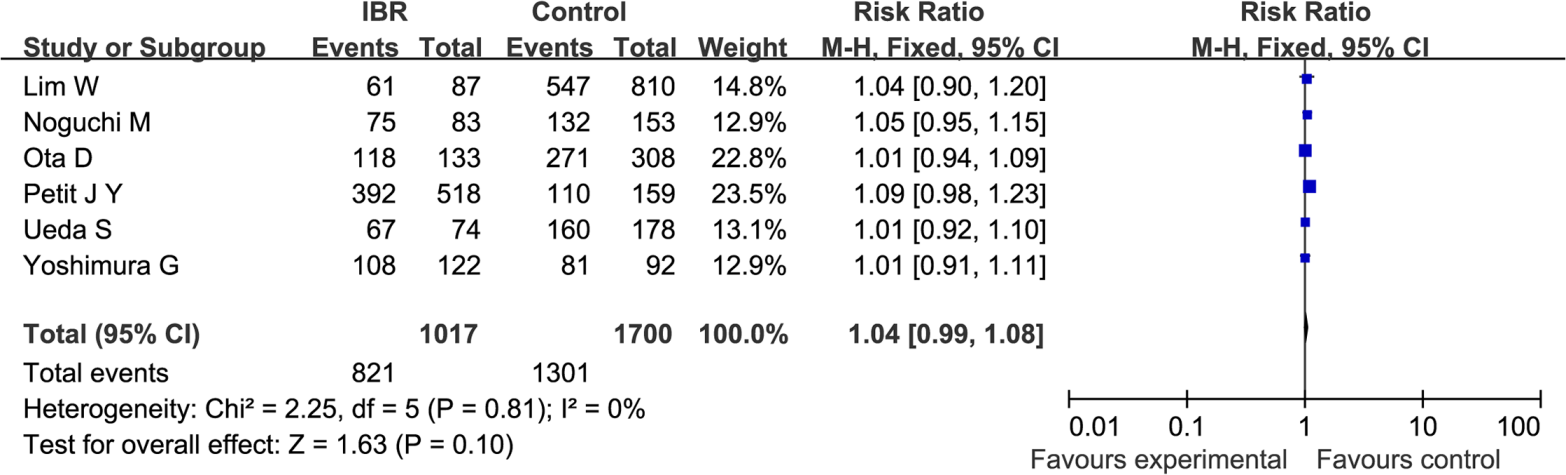
A Forest plot of the pooled RR of DFS for the IBR and Control groups.

DFS was reported by 6 studies that enrolled a total of 1017 cases and 1700 controls. The heterogeneity among the studies was not significant (Q statistic: *P* = 0.81; I^2^ = 0%). The pooled RR was 1.04 (95%CI: 0.99, 1.08), indicating that IBR was not associated with an increased risk in patients’ DFS (*P* = 0.10; Fig 4).

**Fig 5:**
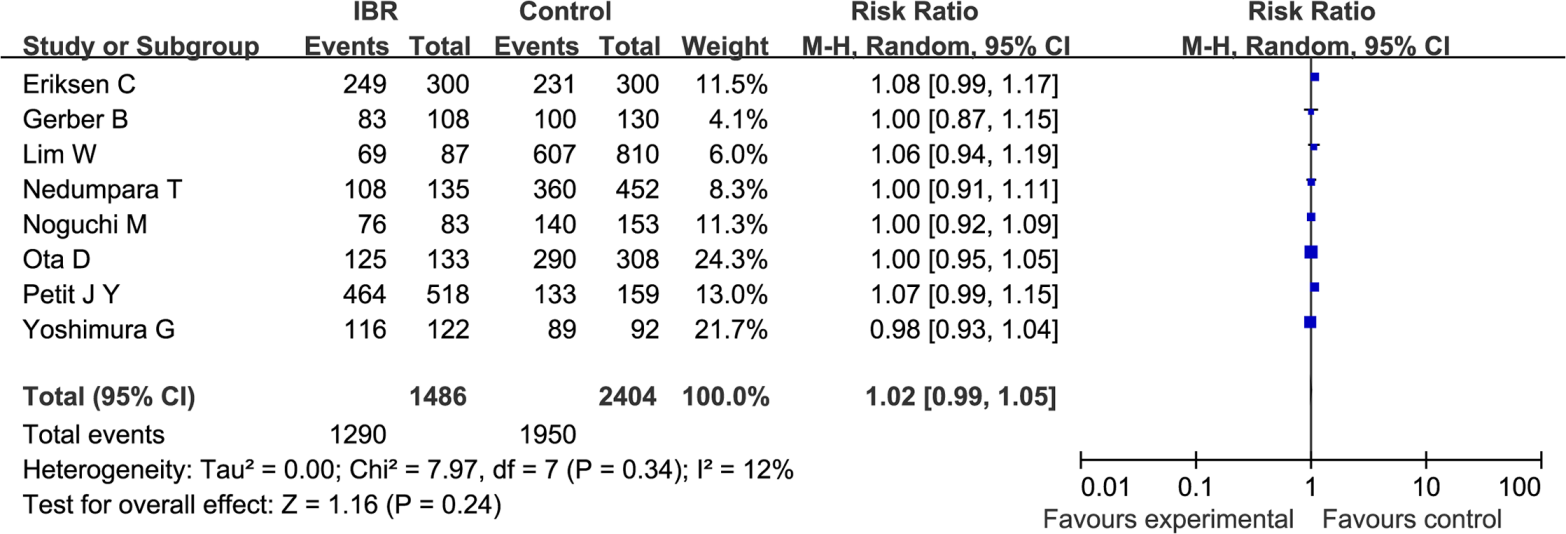
A Forest plot of the pooled RR of OS for the IBR and Control group

OS was reported by 8 studies that enrolled a total of 1486 cases and 2404 controls. There was no significant heterogeneity among the studies (Q statistic: P = 0.34; I^2^ = 12%). The pooled RR, which was 1.02 (95%CI: 0.99, 1.05; *P* = 0.24), demonstrates that IBR had no impairment on prognosis of patients with invasive breast cancer (Fig 5)

In sensitive analysis, the results for recurrence, DFS, and OS were consistent in each single exclusion analysis (Table 4).

**Table 4:** Sensitive analysis by excluding each single study.

Results Excluded study	Recurrence RR(95%CI) and P value	DFS RR(95%CI) and P value	OS RR(95%CI) and P value
**Noguchi[19]**	0.90(0.79, 1.03), P = 0.11	0.99(0.93, 1.05),P = 0.71	1.02(0.99, 1.06), P = 0.22
**Yoshimura[20]**	0.89(0.78, 1.02), P = 0.09	1.00(0.94, 1.05), P = 0.91	1.03(0.99, 1.06), P = 0.10
**Murphy[21]**	0.89(0.78, 1.02), P = 0.09		
**Petit[22]**	0.93(0.80, 1.07), P = 0.30	1.02(0.98, 1.07), P = 0.39	1.01(0.98, 1.04), P = 0.58
**Ueda[23]**	0.90(0.78, 1.02), P = 0.10	1.00(0.94, 1.05), P = 0.90	
**McCarthy[24]**	0.92(0.80, 1.07), P = 0.28		
**Gerber[25]**	0.90(0.78,1.02), P = 0.11		1.02(0.99, 1.05), P = 0.24
**Min[26]**	0.90(0.78, 1.02), P = 0.11		
**Lim[27]**	0.89(0.78,1.01), P = 0.08	0.99(0.94, 1.04), P = 0.69	1.02(0.98, 1.05), P = 0.34
**Eriksen[28]**	0.91(0.78,1.06), P = 0.24		1.01(0.98, 1.04), P = 0.56
**Nedumpara[29]**	0.89(0.78, 1.02), P = 0.09		1.02(0.99, 1.06), P = 0.24
**Reddy[30]**	0.91(0.80, 1.04), P = 0.17		
**Lee[31]**	0.87(0.76,1.01), P = 0.06		
**Ota[32]**	0.89(0.78, 1.02), P = 0.10	0.99(0.93, 1.06), P = 0.86	1.02(0.99, 1.06), P = 0.17

## Discussion

Mastectomy remains the primary treatment for patients with breast cancer. Studies have shown that in recent years, the proportion of patients who choose mastectomy is increasing compared to breast-conserving surgery, and patients are more likely to be advised to select mastectomy for better control of local recurrence[33,34]. IBR after surgery can benefit patients, and an increasing number of patients now opt to undergo this procedure. However, its application is still limited. There are several possible reasons for this limitation, such as concerns about how the surgery will affect future treatments, the fact that breast reconstruction is not a high priority, or lack of knowledge about this type of surgery.

Our meta-analysis shows that IBR is not associated with an increased risk of recurrence (*P* = 0.14). There was no significant publication bias among the included studies, so bias had little effect on the pooled result. A meta-analysis by Gieni et al. (2012) examined the local recurrence of breast cancer among patients who received IBR. The pooled result of this analysis showed no evidence for an association between increased frequency of local recurrence and IBR after mastectomy compared with mastectomy alone[35]. These findings are similar to our own in regards to the rate of recurrence.

Meanwhile, there were no significant differences in DFS or OS between the patients who underwent IBR and the control patients, IBR did not have adverse effect on the prognosis of patients with breast cancer. Some studies even have a result that IBR was associated with an improved prognosis[36–38]. Bezuhly et al used data from the SEER registries to study breast cancer-specific survival in women who underwent unilateral mastectomy with or without IBR. The authors observed improved survival among patients in the IBR group compared with that of patients who underwent mastectomy alone (hazard ratio [HR] = 0.74; 95%CI, 0.68, 0.80)[36]. Studies by Agarwal S and Agarwal J[37,38], one of which was a multivariate analysis that controlled for the demographic and oncologic covariates of 51702 patients[37], showed that patients treated with mastectomy and reconstruction had a significantly lower risk of death than did patients treated with mastectomy only (HR: 0.62, *P*<0.001 and 0.73, *P*<0.0001). The underlying reasons for this phenomenon may be due to direct physiological or immunological effects[39]. However, another possibility is that patients who receive IBR may be more invested in their care. Therefore, these patients may be more likely to pursue oncological or general healthcare, and this behavior may translate to higher survival[37]. Morrow et al have shown that patients with a family income of $40 000 or more were more likely to undergo reconstruction than were patients with an income of less than $40 000[40]. Women with higher incomes may experience improved survival after breast cancer because of better access to cancer care and treatment.

Our study is a systematic review of the available literature that examined overall recurrence/DFS/OS in patients undergoing mastectomy with or without IBR. There are limitations inherent in retrospective reviews, including bias that occurs when designing the review, and this may be a limitation of our study. We limited our search to manuscripts published in English; thus, we may have missed some studies published in other languages. Similarly, the key words that we used may have affected the search. Although overall recurrence included both local and systemic recurrence, not all of the published studies reported data for this outcome. In addition, DFS and OS were also not included in all studies; this is a third limitation of our meta-analysis. We searched for studies with inclusive criteria; such a restriction may have an effect on the final results by excluding studies that had fewer patients, shorter follow-up time or lacked a comparison group. These restrictions may also have introduced a limitation to our study. Finally, some confounding patient factors that affect prognosis, such as tumor stage, patient age, and patient social economic status cannot be controlled in retrospective studies and may also have effect on the results.

Meta-analysis is usually applied to observational studies. One report indicates that the tendency towards publication bias is greater with observational studies than with randomized clinical trials[41]. Publication bias should always be evaluated to assess the credibility of any meta-analysis. Other studies have found that a smaller sample size or negative results in a published study are more likely to lead to publication bias and that the selective publication of meaningful or important discoveries will affect the reliability of the pooled results of a meta-analysis[42,43]. For this study, we evaluated publication bias by constructing a funnel plot, which demonstrated that there was no apparent publication bias in our study. To test the reliability of these meta-analysis results, we need to do a more sensitive analysis. There are different methods to accomplish this task. We used the method of excluding each single study in series, and we found that the results for recurrence, DFS, and OS were consistent in each single exclusion analysis, Thus, we are confident that the results of this study are reliable.

In this meta-analysis, we found no evidence to support the hypothesis that IBR increases the risk of postoperative recurrence and death. Therefore, this procedure can be a safe and rational choice.

## Conclusion

We examined the effects of immediate breast reconstruction on patient prognosis by conducting a meta-analysis. Our results suggest that there are no significant differences in overall recurrence, DFS, or OS between patients who have undergone IBR after mastectomy and those who were treated with mastectomy only. More high-quality evidence is needed in this field to allow clinicians to make better choices regarding treatment regimens.

## Supporting Information

S1 PRISMA Checklist PRISMA checklist. (DOC)
